# Milk Composition and Production Efficiency within Feed-To-Yield Systems on Commercial Dairy Farms in Northern Ireland

**DOI:** 10.3390/ani12141771

**Published:** 2022-07-11

**Authors:** Aimee-Louise Craig, Alan W. Gordon, Gregory Hamill, Conrad P. Ferris

**Affiliations:** 1 Agri-Food and Bioscience Institute Hillsborough, Large Park, Hillsborough BT26 6DR, UK; gregory.hamill@daera-ni.gov.uk (G.H.); conrad.ferris@afbini.gov.uk (C.P.F.); 2 Agri-Food and Bioscience Institute Newforge, Newforge Lane, Belfast BT9 5PX, UK; alan.gordon@afbini.gov.uk

**Keywords:** dairy cattle, feed-to-yield, milk composition, concentrate use efficiency, nitrogen efficiency

## Abstract

**Simple Summary:**

Feed-to-yield concentrate allocation systems seek to improve precision by targeting more nutrients to dairy cows with the greatest nutrient requirements. This study investigated the effect of offering concentrates on a feed-to-yield basis to housed cows on the relationships between milk yield, intake, milk composition and production efficiency during the first five months of lactation. Performance data for individual cows were collected from 26 farms in Northern Ireland, and intakes were subsequently calculated. Total dry matter intake increased with increasing milk yield. Cows with higher milk yields produced milk with a lower fat and protein concentration, likely due to a combination of cow genetics, diet, and a ‘dilution’ effect associated with yield. The reduction in milk fat and milk protein concentration with increasing milk yield is particularly important for farmers supplying milk for processing, as milk pricing mechanisms within these supply contracts normally include bonuses or deductions determined by milk composition. Cows with greater milk yields had improved nitrogen and energy use efficiency but were offered more concentrates per kilogram of energy-corrected milk produced.

**Abstract:**

This study examined the relationships between milk yield and diet composition, nutrient intakes, milk composition, and feed use efficiency when concentrates were offered using a feed-to-yield (FTY) approach. The study was conducted on 26 dairy farms in Northern Ireland. Cows (*n* = 3471) were fully housed and were offered concentrates on an FTY basis. Individual cow genetic information was obtained for 18 herds. Concentrate intakes of individual cows were either obtained from the farms or calculated, while milk yield and milk composition data were obtained from test-day milk recording. Mean test-day milk yields during months 2 to 5 post-calving were calculated for each cow, and cows within each lactation were placed into one of six equal-sized milk yield (kg/cow/day) groups. Diet effects and performance responses to milk yield groups were tested for linear and quadratic effects. Total dry matter intakes increased with increasing milk yield. Milk fat and milk protein concentration declined as milk yield increased, which could be attributed in part to genetics and diet. As milk yield increased, nitrogen and energy use efficiency was improved. However, concentrates offered per kg of energy-corrected milk also increased at higher milk yields, indicating an increased reliance on concentrates for these cows.

## 1. Introduction

The use of Holstein sires with a high transmitting ability for milk volume has contributed to increased milk yields per cow in many countries [1,2]. However, in early lactation, dry matter intake (DMI) often does not increase at the same rate as milk production [3], and cows frequently enter a period of negative energy balance (EB). In an attempt to reduce the extent and duration of this negative EB and to support these greater milk yields, the quantity of concentrates offered to dairy cows has increased in many countries. For example, within Northern Ireland (NI), annual concentrate inputs increased from 1.8 to 2.7 t/cow between 2004 and 2020 (DAERA statistics).

A wide range of concentrate feeding approaches are adopted on farms, including mixing concentrates with the forage component of the diet as part of a total mixed ration (TMR) or presenting them separately from the forage via in-parlour or out-of-parlour (OPF) feeding systems, or a combination of these approaches. With systems involving in-parlour or OPF feeding systems, concentrates are often offered on a feed-to-yield (FTY) basis, and this approach is now commonplace on many farms. Nevertheless, a number of recent studies had demonstrated that average performance was relatively unaffected when the same quantity of concentrates was offered over the winter using either ‘flat-rate’ or FTY approaches [4,5]. However, most studies involving FTY systems do not examine the performance of individual cows, which is critical given that an FTY approach increases the range of concentrate intakes and performance levels within a herd. While increased concentrate feeding levels drive milk production, increased concentrate intake is known to reduce forage DMI through the substitution effect, while high concentrate intake are known to depress milk fat [6,7]. For example, a study at the Agri-Food and Bioscience Institute (AFBI; C.P. Ferris, Unpublished data) suggested that when concentrates were offered on an FTY basis, milk fat concentrations of the highest yielding cows were substantially reduced, reducing the economic value of milk produced by these cows.

While cow performance associated with FTY approaches has been periodically examined within a research setting [4,8,9], few studies have specifically examined performance within FTY systems on commercial farms. The objectives of this study were to investigate how FTY systems operate in practice on commercial farms in NI and to examine the relationship between milk yield and DMI, milk composition and production efficiency. Specifically, the study sought to identify if a reduction in milk fat concentration was observed in cows offered higher levels of concentrates, and if so, how much of the reduction could be attributed to cow genetics.

## 2. Materials and Methods

### 2.1. Farm Selection and Cow Details

The study and its objectives were advertised through the local press and farmers were invited to apply if they met the following criteria: located in NI, predominantly Holstein-Friesian herds, at least 50 Holstein-Friesian cows in the herd due to calve between August 2018 and February 2019, an annual milk yield/cow in excess of 6500 kg during the previous year, participating in an official milk recording scheme, offering concentrates on an FTY basis, and willingness to provide AFBI with the required information. Thirty-one farms were selected to participate in the study, with data from five farms subsequently excluded (details in ‘Statistical Analysis’). During the year prior to this study, the remaining 26 farms had an average herd size, concentrate feed level (t/cow/year) and 305-day milk yield of 195 cows (range: 80–500), 2.9 t (range: 1.6–4.0) and 8816 kg (range: 6900–11,643), respectively. Across the 26 farms included in the final analysis, 3471 cows (average lactation number, 2.7: range: 1–15; s.d. 1.8) which calved between 1 August 2018 and 28 February 2019, were enrolled on the study. Dairy herds on 18 of the participating farms were pedigree registered. 

### 2.2. Concentrate Feeding Systems Adopted

On 19 of the 26 farms, cows were offered a ‘basal ration’, comprising a mixture of forage and concentrate ingredients, prepared using a mixer wagon with additional concentrates offered using either an in-parlour feeding system (*n* = 14) or an OPF system (*n* = 1), or both (*n* = 4). On the remaining seven farms, cows were offered silage either through a mixer wagon (*n* = 1), via blocks of silage placed along a feed barrier (*n* = 5), or cows had access to silage directly at the silo face (*n* = 1: ‘self-feeding’) with concentrates offered using either an in-parlour feeding system (*n* = 2) or both in-parlour and OPF feeding systems (*n* = 5). On all farms, the concentrate component of the diet offered on an FTY basis was offered using either in-parlour or OPF feeding systems.

### 2.3. Data Collection

Each farm was visited on 5–6 occasions, at approximately 6–8 week intervals, between September 2018 and May 2019. During each visit, detailed information on feeding practices was collected, including information on all forages offered (type and quantity), details and quantities of other diet ingredients, information on each type of concentrate offered and how it was offered, feeding management practices, maximum concentrate levels, dates of diet changes, and details of turnout to grass (herd/group basis or individual cow basis). In addition, a sample of each forage type and each concentrate type offered was taken during each visit. Concentrate samples were dried at 60 °C for 48 h, milled through a 0.85 mm sieve, and subsequently analysed for nitrogen (N) and starch. Nitrogen concentrations were determined using the Dumas method, while starch concentrations were determined using a Megazyme kit (Megazyme International, Bray, Ireland). The metabolisable energy (ME) content of all concentrates was assumed as 13 MJ/kg DM (FeedByte^®^, SRUC, Edinburgh, UK), while the ME, N and starch content of ‘straights’ and by-product ingredients (which were offered on a small number of farms: *n* = 3) were obtained from FeedByte (FeedByte^®^, SRUC, Edinburgh, UK). The composition of grass silages (volatile corrected DM, crude protein (CP), neutral detergent fibre (NDF), ME, pH, ammonia nitrogen, and predicted acid load), maize silages and whole crop silages (NDF, DM, ME, starch and CP) were predicted using NIRS, as described by Park et al. [10].

### 2.4. Milk Yield and Composition

All farms participated in a formal milk recording scheme which provided data on individual cow test-day milk yields, milk fat and milk protein concentrations. Milk recording was conducted monthly on 21 farms, while on the remaining five farms, milk recording was conducted every 6–8 weeks (an average of 7.7 recordings per farm (range 6–8) during the study). Data obtained at each milk recording was used to determine the gross energy (GE) content of the milk produced by each cow, as follows: [11]

Milk GE (MJ/kg) = 0.0376 × Fat (g/kg) + 0.0209 × Protein (g/kg) + 0.948

Energy corrected milk (ECM) yield (kg/day) was then calculated assuming the GE content of 1 kg ‘standard milk’ to be 3.1 MJ/kg (i.e., for milk containing 40 g/kg fat, 32 g/kg crude protein, and 48 g/kg lactose, as described by Muñoz et al. [12], according to the following equation:

ECM (kg/d) = (milk yield (kg/d) × GE (MJ/kg))/3.1.

### 2.5. Dry Matter Intake

At each milk recording occasion on each farm, the total DMI of each individual cow was estimated using the following equation, which was developed specifically for cows offered concentrates on an FTY basis: [13]

DMI (kg/day) = 11.032 + (0.554 × lactation number) + (0.343 × ECM kg) + (−3.194 × Fat:Protein) + (0.107 × week-in-milk)

Having estimated total DMI, the DMI of each component of the diet was subsequently obtained or calculated for each individual cow. On 11 farms, the quantity of concentrates offered to each individual cow via in-parlour or OPF were downloaded from the feeding system software, and the average concentrate intake for the seven-day period prior to the date of milk recording was determined. Intakes of concentrates via in-parlour and OPF feeding systems on the remaining 15 farms were calculated for each individual cow using milk yield data and the feeding assumptions in place on the farm at the time of milk recording. Specifically, this required the ‘Maintenance Plus’ (M+) value, namely the yield of milk (in addition to the cows’ maintenance energy requirements) that the basal ration or forage offered was assumed by the farmer to support, and the concentrate feed rate (kg concentrate offered/kg of milk produced in excess of the M+ value), as follows:

Concentrate intake via in-parlour or OPF system (kg/day) = (recorded milk yield−M+ value) × concentrate feed rate

Fresh concentrate intakes were converted to a DM basis by assuming a DM of 88% for all concentrates. Concentrates offered through either the in-parlour and OPF feeding systems were then deducted from the estimated total daily DMI for each individual cow, and the remainder of the daily DMI was, therefore, either forage or a mixture of forage and concentrates, depending on the diets offered on each individual farm. In the case of the latter, the recorded fresh weight ratios of individual components placed in the mixer wagon and subsequently DM ratios (based on measured forage DM concentrations) were determined. The DM ratios of individual ration ingredients were used to apportion the remainder of the daily DMI to individual diet components. Daily intakes of N, starch and ME from the individual components of the diet were subsequently calculated. 

### 2.6. Genetic Information

For the 18 herds that were pedigree registered, the genetic information (£PLI, PTA (Predicted Transmitting Ability) Milk (kg), PTA Fat%, PTA Protein%) for each cow was obtained from the December 2019 dairy herd genetic reports (available online from: https://ahdb.org.uk/knowledge-library/dairy-herd-genetic-reports, accessed on 3 May 2019). The mean PTA for £PLI, milk yield kg, milk fat% and milk protein% were £173 (range: £−229 to £625), 103 kg (range: −714 kg to 988 kg), 0.06% (range: −0.25% to 0.38%) and 0.05% (range: −0.12% to 0.20%), respectively with an overall reliability of 62% (range: 21% to 83%).

### 2.7. Statistical Analysis

Data from five of the 31 herds selected to participate in the study were excluded from the analysis due to: concern over the quality of the individual cow data available (*n* = 2), very low herd concentrate inputs (*n* = 2) and absence of a true FTY approach at individual cow level (*n* = 1). The data analysed were restricted to months 2–5 of lactation, encompassing a period when cows were fully housed and when concentrates were offered on an FTY basis (most herds did not adopt an FTY approach until at least 3 weeks post-calving). Data were analysed separately for each of lactations 1, 2, 3 and 4+. Within each lactation, the mean test-day milk yield for each individual cow during months 2–5 post-calving was calculated, and cows were then ranked by milk yield and sub-divided into one of six equal-sized milk yield groups based on percentiles. Each milk yield ‘group’ became the ‘treatment group’, and individual cow performance data was analysed using a linear mixed model (REML estimation method) with farm fitted as a random effect and milk yield group as a fixed effect using the following model where Y_ij_ is the response from animal j in farm i on the measured variable Y; m is the overall constant; MilkYield_t_ is the fixed effect of milk yield where t is the group associated with animal j on the farm i; Farm_i_ is the random effect of farm i; E_ij_ is the random error for animal j within farm i.:

Y_ij_ = m + MilkYield_t_ + Farm_i_ + E_ij_

Milk yield was treated as an independent variable, with all other analysed variables treated as a response. In addition, milk yield was assessed for linear and quadratic trends. In all cases, the adequacy of the models was assessed by visual inspection of the appropriate residual plots. All analyses were carried out using the statistical software package GenStat 20th edition (VSN International Limited, Oxford, UK).

## 3. Results

### 3.1. Approaches to FTY and Composition of Diet Components

**Figure 1:**
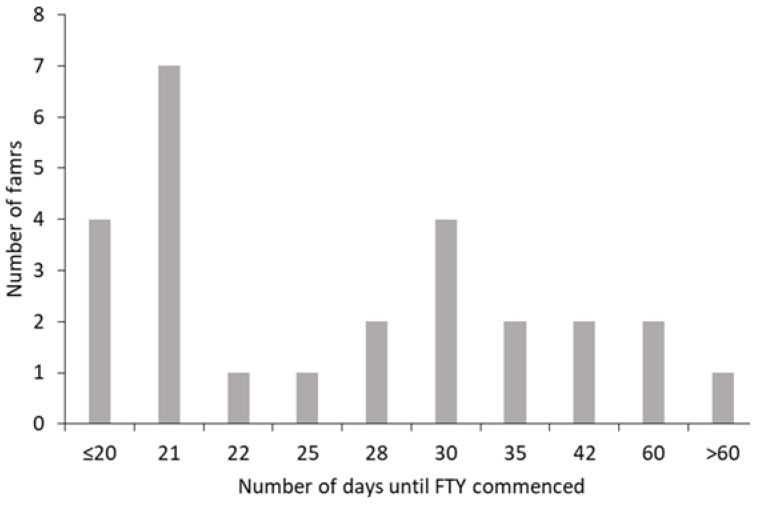
Duration of concentrate build-up periods before cows were offered concentrates on an FTY basis.

All farms adopted a concentrate ‘build-up’ period before FTY commenced (range, 10–100 days), although the majority of farms had adopted an FTY approach by day 30 of lactation (Figure 1). At the time when FTY was adopted, total concentrate intakes averaged 9.7 kg/d (range: 5–16 kg) for multiparous cows and 7.8 kg/d (range: 3.5–14 kg) for primiparous cows. Across the 26 farms, the average M+ value adopted during months 2–5 post-calving was 20 (range: 8–30) kg and 17 (range: 6–28) kg of milk/day for cows and heifers, respectively. Concentrates offered to support milk produced in excess of the M+ value were offered at a rate of 0.45 kg/kg milk on 19 farms, while three farms used a feed rate of 0.40 kg/kg milk, and the remaining four farms used values of 0.42, 0.43, 0.50 and 0.52 kg/kg milk.

Grass silage was the predominant forage in the diet on all farms. While the quality of grass silage offered varied between farms (Table 1), the average nutritive value was good, having a mean DM, crude protein and ME content of 318 g/kg, 139 g/kg DM and 11.2 MJ/kg DM, respectively. In addition to grass silage, 17 farms offered alternative forages, either fermented whole-crop silage (*n* = 10), maize silage (*n* = 3), or both whole-crop silage and maize silage (*n* = 4). Concentrates offered, both on an FTY basis and as part of a basal diet had a wide range of CP and starch concentrations (Table 1).

### 3.2. Relationships between Milk Yield Group and DMI, Nutrient Intakes, Diet Composition, Milk Production and Feed Use Efficiency

Dry matter intakes, diet composition, nutrient intakes, milk production and efficiency values within each milk yield group are presented for lactations 1, 2, 3 and 4+ in Table 2, Table 3, Table 4 and Table 5, respectively. Total concentrate DMI showed a quadratic increase with increasing milk yield group in first lactation cows (*p* = 0.003) and a linear increase (*p* < 0.001) in multiparous cows. Total DMI showed a positive linear response with increasing milk yield (*p* < 0.001) within all lactations. There was no significant effect of the milk yield group on forage DMI in lactation 3. Forage DMI showed a positive quadratic response to milk yield group within lactation 1 (*p* = 0.001), a positive linear response within lactation 2 (*p* = 0.029) and a negative linear response in lactation 4+ (*p* < 0.001). 

The concentrate proportion of the diet showed a linear increase with increasing milk yield group in lactation 1 and 2 (*p* < 0.001), and a quadratic increase in lactation 3 (*p* = 0.014) and 4+ (*p* = 0.005). The proportion of alternative forages in the diet decreased linearly with increasing milk yield group in all lactations (*p* < 0.001). Total starch, N and ME intake increased as the milk yield group increased across all lactations (*p* < 0.001; Table 2, Table 3, Table 4 and Table 5). These were mostly linear responses, except starch intake within lactation 1 (*p* < 0.001) and 3 (*p* = 0.019), and total ME intake in lactation 4+ (*p* = 0.042), which followed quadratic responses. 

Total diet starch and CP percentage increased with increasing milk yield group in all lactations, with the response for starch percentage quadratic (*p* < 0.05), except within lactation 3 where a linear response was observed (*p* < 0.001). Crude protein percentage of the total diet followed a linear response (*p* < 0.001) in lactations 1 and 2, and a quadratic response (*p* = 0.005) in lactation 3 and 4+. Effect of milk yield group on total diet ME concentration was not significant in lactation 1, but showed a quadratic increase in lactation 2 (*p* = 0.047) and 4+ (*p* = 0.037) and a linear increase in lactation 3 (*p* = 0.001).

Milk fat percentage showed a linear decline with increasing milk yield group in all lactations (*p* < 0.001; Table 2, Table 3, Table 4 and Table 5), while milk protein percentage showed a linear (*p* < 0.001) decrease in all lactations, with the exception of lactation 2 where a quadratic response (*p* = 0.017) was observed. Fat plus protein yield and ECM yield increased (*p* < 0.001) with increasing milk yield group, with the response linear in all lactations except lactation 4+ where the responses were quadratic. 

Nitrogen use efficiency (NUE; milk N/N intake) showed a quadratic increase (*p* < 0.001 in lactations 1, 2 and 4+, and *p* = 0.012 in lactation 3) with an increase in the milk yield group. Milk energy/ME intake showed a quadratic increase (*p* < 0.001 in lactations 1, 3 and 4+, and *p* = 0.034 in lactation 2) with an increase in the milk yield group, while ECM/DMI showed a quadratic increase across all lactations (*p* < 0.001). Concentrate DMI (kg) offered per kg milk yield showed a quadratic decrease (*p* = 0.009) with an increase in the milk yield group in first lactation cows but was unaffected by the milk yield group in subsequent lactations. In contrast, concentrate DMI (kg) offered per kg ECM yield showed a quadratic increase with an increase in the milk yield group in lactation 1, and a linear increase in lactation 2 (*p* < 0.001), 3 (*p* = 0.003) and 4+ (*p* < 0.001).

### 3.3. Relationship between Cow Profile and Genetic Merit

Irrespective of the milk yield group, there were general trends for PLI and PTA for milk yield to decrease with increasing lactation number (Table 6). PLI showed a positive linear increase with increasing milk yield group (*p* = 0.038 in lactation 1, and *p* < 0.001 in lactations 2, 3 and 4+). In lactations 1, 3 and 4+ PTA for milk yield showed a positive linear response (*p* < 0.001), while the response was quadratic in lactation 2 (*p* = 0.004). However, PTA for milk fat percentage showed a decreasing linear response with increasing milk yield group in Lactations 1 (*p* = 0.003) and 3 (*p* < 0.001) and a decreasing quadratic response in lactation 2 (*p* = 0.003) and 4+ (*p* = 0.036). Likewise, PTA for milk protein percent decreased in all lactations with increasing milk yield group, with the response linear in lactation 1 (*p* = 0.002), 3 and 4+ (*p* = 0.001), and quadratic in lactation 2 (*p* = 0.009). 

**Table 2:** Dry matter intakes, nutrient intakes and diet composition, milk production, and efficiency values within each milk yield group for first lactation cows.

		Milk Yield Group (kg/cow/day)							*p*-Value	
	Range	13.8–23.1	23.1–25.6	25.6–27.9	27.9–30.7	30.7–34.2	34.2–47.7	SED	Linear	Quadratic
	Average	20.6	24.4	26.7	29.3	32.4	37.6			
Dry matter intake (DMI)										
	Concentrate offered on a feed-to-yield basis (kg)	3.2	3.9	4.5	5.2	6.1	7.7	0.10	<0.001	<0.001
	Total concentrate (kg/day)	6.6	7.5	8.2	8.9	9.8	11.5	0.09	<0.001	0.003
	Total forage (kg/day)	9.7	10.1	10.1	10.2	10.2	10.3	0.09	<0.001	0.001
	Total (kg/day)	16.3	17.6	18.3	19.1	20.1	21.8	0.08	<0.001	0.735
Diet composition and nutrient intakes										
	Concentrate as % total DMI	40.3	42.6	44.6	46.6	49.0	52.6	0.30	<0.001	0.110
	‘Alternative forages’ as % total DMI	9.9	9.2	8.7	8.3	8.3	7.6	0.29	<0.001	0.160
	Intake of starch from concentrates (g/d)	1564	1784	1943	2117	2333	2760	22.3	<0.001	<0.001
	Total starch intake (g/d)	2013	2225	2399	2555	2804	3262	26.8	<0.001	<0.001
	Starch % of total diet	12.4	12.6	13.1	13.3	13.8	14.6	0.12	<0.001	0.036
	Intake of nitrogen from concentrates (g/d)	217	247	267	292	320	372	2.9	<0.001	0.003
	Total nitrogen intake (g/day)	417	454	476	501	529	582	2.4	<0.001	0.303
	Crude protein % of total diet	16.0	16.1	16.3	16.4	16.5	16.8	0.04	<0.001	0.459
	Intake of ME from concentrate (g/d)	87	99	107	118	129	150	1.2	<0.001	0.006
	Total ME intake (MJ/day)	197	213	220	234	244	265	1.7	<0.001	0.966
	ME concentration of total diet (MJ/kg DM)	12.1	12.1	12.1	12.2	12.2	12.2	0.09	0.235	0.998
Milk production										
	Milk fat (%)	4.36	4.28	4.23	4.13	4.01	3.87	0.049	<0.001	0.461
	Milk protein (%)	3.36	3.31	3.26	3.22	3.17	3.14	0.021	<0.001	0.075
	Fat + Protein yield (kg/day)	1.60	1.86	2.01	2.17	2.33	2.64	0.021	<0.001	0.078
	Energy corrected milk yield (kg/day)	21.9	25.7	27.8	30.1	32.6	37.1	0.26	<0.001	0.098
Feed use efficiency (daily basis)										
	Milk nitrogen output/N intake (kg/kg)	0.26	0.28	0.29	0.30	0.31	0.32	0.002	<0.001	<0.001
	Gross energy output in milk/ME intake (MJ/MJ)	0.35	0.38	0.39	0.41	0.42	0.44	0.003	<0.001	<0.001
	ECM/DMI (kg/kg)	1.34	1.46	1.52	1.57	1.62	1.70	0.009	<0.001	<0.001
	Concentrate DMI/milk yield (kg/kg)	0.31	0.30	0.30	0.30	0.30	0.30	0.003	0.019	0.009
	Concentrate DMI/ECM yield (kg/kg)	0.30	0.29	0.29	0.30	0.30	0.31	0.004	<0.001	<0.001

‘Alternative forages’ comprised whole crop maize, wheat or oat silage: DMI, dry matter intake; ME, metabolisable energy; ECM, energy corrected milk.

**Table 3:** Dry matter intakes, nutrient intakes and diet composition, milk production, and efficiency values within each milk yield group for second lactation cows.

		Milk Yield Group (kg/cow/day)							*p*-Value	
	Range	13.7–28.5	28.5–32.3	32.3–35.6	35.6–38.7	38.7–43.0	43.0–57.3	SED	Linear	Quadratic
	Average	25.5	30.6	34.0	37.0	40.8	47.2			
Dry matter intake (DMI)										
	Concentrate offered on a feed-to-yield basis (kg)	4.0	5.4	6.2	7.0	8.1	10.0	0.14	<0.001	0.074
	Total concentrate (kg/day)	7.9	9.4	10.2	11.1	12.1	14.0	0.12	<0.001	0.404
	Total forage (kg/day)	11.1	11.2	11.3	11.3	11.3	11.3	0.12	0.029	0.227
	Total (kg/day)	18.9	20.5	21.5	22.4	23.4	25.3	0.11	<0.001	0.647
Diet composition and nutrient intakes										
	Concentrate as % total DMI	41.6	45.5	47.6	49.4	51.6	55.2	0.30	<0.001	0.211
	‘Alternative forages’ as % total DMI	9.0	8.7	8.0	8.2	7.4	6.8	0.31	<0.001	0.488
	Intake of starch from concentrates (g/d)	1891	2251	2440	2643	2888	3350	29.5	<0.001	0.125
	Total starch intake (g/d)	2390	2755	2944	3173	3397	3897	33.5	<0.001	0.082
	Starch % of total diet	12.5	13.4	13.6	14.1	14.4	15.0	0.13	<0.001	0.015
	Intake of nitrogen from concentrates (g/d)	258	306	333	360	394	452	3.9	<0.001	0.571
	Total nitrogen intake (g/day)	486	536	564	593	627	683	3.4	<0.001	0.615
	Crude protein % of total diet	16.1	16.3	16.4	16.6	16.8	16.9	0.001	<0.001	0.101
	Intake of ME from concentrate (g/d)	104	124	135	146	160	184	1.5	<0.001	0.626
	Total ME intake (MJ/day)	228	252	265	276	289	314	2.0	<0.001	0.084
	ME concentration of total diet (MJ/kg DM)	12.1	12.3	12.4	12.4	12.4	12.5	0.07	<0.001	0.047
Milk production										
	Milk fat (%)	4.41	4.22	4.11	4.03	3.94	3.74	0.062	<0.001	0.674
	Milk protein (%)	3.48	3.37	3.29	3.25	3.16	3.12	0.026	<0.001	0.017
	Fat + Protein yield (kg/day)	2.05	2.35	2.54	2.70	2.90	3.24	0.032	<0.001	0.501
	Energy corrected milk yield (kg/day)	27.9	32.3	35.1	37.6	40.7	45.8	0.39	<0.001	0.614
Feed use efficiency (daily basis)										
	Milk nitrogen output/N intake (kg/kg)	0.29	0.30	0.31	0.32	0.32	0.34	0.002	<0.001	<0.001
	Gross energy output in milk/ME intake (MJ/MJ)	0.38	0.40	0.42	0.43	0.44	0.46	0.004	<0.001	0.034
	ECM/DMI (kg/kg)	1.46	1.57	1.63	1.68	1.73	1.81	0.012	<0.001	<0.001
	Concentrate DMI/milk yield (kg/kg)	0.30	0.30	0.30	0.30	0.30	0.30	0.004	0.475	0.101
	Concentrate DMI/ECM yield (kg/kg)	0.28	0.29	0.29	0.29	0.30	0.31	0.004	<0.001	0.456

‘Alternative forages’ comprised whole crop maize, wheat or oat silage: DMI, dry matter intake; ME, metabolisable energy; ECM, energy corrected milk.

**Table 4:** Dry matter intakes, nutrient intakes and diet composition, milk production, and efficiency values within each milk yield group for third lactation cows.

		Milk Yield Group (kg/cow/day)							*p*-Value	
	Range	16.7–32.0	32.0–35.3	35.3–38.5	38.5–41.7	41.7–46.0	46.0–63.7	SED	Linear	Quadratic
	Average	27.7	33.9	37.0	40.0	43.9	50.8			
Dry matter intake (DMI)										
	Concentrate offered on a feed-to-yield basis (kg)	4.6	6.2	7.3	8.0	9.0	11.0	0.16	<0.001	0.610
	Total concentrate (kg/day)	8.6	10.4	11.5	12.2	13.2	15.3	0.14	<0.001	0.972
	Total forage (kg/day)	11.6	11.7	11.5	11.9	11.8	11.7	0.13	0.203	0.292
	Total (kg/day)	20.2	22.1	23.0	24.1	25.0	27.0	0.15	<0.001	0.353
Diet composition and nutrient intakes										
	Concentrate as % total DMI	42.7	47.2	49.8	50.7	52.8	56.6	0.30	<0.001	0.014
	‘Alternative forages’ as % total DMI	9.5	8.5	7.9	7.8	7.3	6.7	0.34	<0.001	0.191
	Intake of starch from concentrates (g/d)	2091	2490	2727	2890	3130	3649	37.7	<0.001	0.150
	Total starch intake (g/d)	2640	3011	3244	3435	3652	4206	40.6	<0.001	0.019
	Starch % of total diet	13.0	13.6	14.1	14.2	14.5	15.3	0.14	<0.001	0.884
	Intake of nitrogen from concentrates (g/d)	279	341	373	399	431	496	4.9	<0.001	0.397
	Total nitrogen intake (g/day)	519	581	611	644	674	735	4.7	<0.001	0.106
	Crude protein % of total diet	16.0	16.4	16.6	16.7	16.9	17.1	0.001	<0.001	0.005
	Intake of ME from concentrate (g/d)	114	136	149	159	172	199	1.9	<0.001	0.681
	Total ME intake (MJ/day)	241	267	278	293	304	330	2.6	<0.001	0.358
	ME concentration of total diet (MJ/kg DM)	11.9	12.1	12.1	12.2	12.2	12.3	0.09	0.001	0.533
Milk production										
	Milk fat (%)	4.30	4.19	4.13	4.10	3.85	3.78	0.076	<0.001	0.489
	Milk protein (%)	3.42	3.34	3.24	3.22	3.15	3.06	0.030	<0.001	0.472
	Fat + Protein yield (kg/day)	2.15	2.55	2.74	2.96	3.10	3.49	0.041	<0.001	0.074
	Energy corrected milk yield (kg/day)	29.4	35.2	38.1	41.2	43.6	49.5	0.50	<0.001	0.130
Feed use efficiency (daily basis)										
	Milk nitrogen output/N intake (kg/kg)	0.29	0.31	0.31	0.31	0.32	0.33	0.003	<0.001	0.012
	Gross energy output in milk/ME intake (MJ/MJ)	0.38	0.41	0.43	0.44	0.45	0.47	0.004	<0.001	<0.001
	ECM/DMI (kg/kg)	1.45	1.59	1.65	1.70	1.74	1.83	0.014	<0.001	<0.001
	Concentrate DMI/milk yield (kg/kg)	0.30	0.30	0.31	0.31	0.29	0.30	0.006	0.398	0.825
	Concentrate DMI/ECM yield (kg/kg)	0.29	0.29	0.30	0.30	0.30	0.31	0.006	0.003	0.777

‘Alternative forages’ comprised whole crop maize, wheat or oat silage: DMI, dry matter intake; ME, metabolisable energy; ECM, energy corrected milk.

**Table 5:** Dry matter intakes, nutrient intakes and diet composition, milk production, and efficiency values within each milk yield group for fourth and higher lactation cows.

		Milk Yield Group (kg/cow/day)							*p*-Value	
	Range	16.5–32.1	32.1–36.1	36.1–39.3	39.3–43.0	43.0–47.0	47.0–70.6	SED	Linear	Quadratic
	Average	28.0	34.0	37.9	40.9	44.8	51.1			
Dry matter intake (DMI)										
	Concentrate offered on a feed-to-yield basis (kg)	4.6	6.3	7.5	8.3	9.5	11.3	0.15	<0.001	0.545
	Total concentrate (kg/day)	8.9	10.7	11.8	12.7	13.9	15.7	0.14	<0.001	0.763
	Total forage (kg/day)	12.8	12.6	12.6	12.5	12.5	12.2	0.13	<0.001	0.256
	Total (kg/day)	21.7	23.3	24.4	25.3	26.5	27.9	0.14	<0.001	0.404
Diet composition and nutrient intakes										
	Concentrate as % total DMI	41.3	46.1	48.5	50.4	52.6	56.3	0.30	<0.001	0.005
	‘Alternative forages’ as % total DMI	9.3	8.8	8.3	7.5	7.2	6.2	0.25	<0.001	0.215
	Intake of starch from concentrates (g/d)	2154	2561	2823	3030	3332	3778	35.5	<0.001	0.131
	Total starch intake (g/d)	2730	3128	3388	3572	3878	4335	37.5	<0.001	0.052
	Starch % of total diet	12.4	13.3	13.9	14.1	14.6	15.3	0.13	<0.001	0.030
	Intake of nitrogen from concentrates (g/d)	292	349	387	415	452	506	4.564	<0.001	0.413
	Total nitrogen intake (g/day)	556	609	646	674	710	756	4.3	<0.001	0.16
	Crude protein % of total diet	16.0	16.3	16.5	16.7	16.8	16.9	0.05	<0.001	0.005
	Intake of ME from concentrate (g/d)	118	142	156	167	184	206	1.8	<0.001	0.804
	Total ME intake (MJ/day)	260	284	300	311	328	344	2.9	<0.001	0.042
	ME concentration of total diet (MJ/kg DM)	12.0	12.2	12.3	12.3	12.3	12.4	0.10	0.001	0.037
Milk production										
	Milk fat (%)	4.41	4.19	4.15	4.06	3.98	3.64	0.060	<0.001	0.058
	Milk protein (%)	3.34	3.27	3.22	3.18	3.14	3.04	0.025	<0.001	0.679
	Fat + Protein yield (kg/day)	2.20	2.56	2.80	3.00	3.19	3.43	0.034	<0.001	<0.001
	Energy corrected milk yield (kg/day)	30.2	35.5	39.0	41.9	44.9	48.9	0.42	<0.001	0.002
Feed use efficiency (daily basis)										
	Milk nitrogen output/N intake (kg/kg)	0.27	0.29	0.30	0.30	0.31	0.32	0.002	<0.001	<0.001
	Gross energy output in milk/ME intake (MJ/MJ)	0.36	0.39	0.41	0.42	0.43	0.45	0.004	<0.001	<0.001
	ECM/DMI (kg/kg)	1.39	1.52	1.59	1.65	1.69	1.75	0.013	<0.001	<0.001
	Concentrate DMI/milk yield (kg/kg)	0.31	0.31	0.31	0.31	0.31	0.31	0.004	0.164	0.936
	Concentrate DMI/ECM yield (kg/kg)	0.29	0.30	0.30	0.30	0.31	0.33	0.005	<0.001	0.208

Alternative forages’ comprised whole crop maize, wheat or oat silage: DMI, dry matter intake; ME, metabolisable energy; ECM, energy corrected milk.

**Table 6:** Profitable Lifetime Index (PLI) and Predicted Transmitting Ability (PTA) for milk yield, milk fat percent and milk protein percent of experimental cows within each milk yield group, within each of lactations 1, 2, 3 and 4+.

		Milk Yield Group							*p*-Value	
		(kg/cow/day)						SED	Linear	Quadratic
Lactation 1		13.8–23.1	23.1–25.6	25.6–27.9	27.9–30.7	30.7–34.2	34.2–47.7			
	PLI £	213	225	236	236	245	257	21.3	0.038	0.823
	Milk yield (kg)	103	129	167	183	220	269	25.5	<0.001	0.931
	Fat (%)	0.06	0.06	0.07	0.05	0.04	0.04	0.010	0.003	0.394
	Protein (%)	0.04	0.04	0.04	0.03	0.03	0.03	0.005	0.002	0.762
Lactation 2		13.7–28.5	28.5–32.3	32.3–35.6	35.6–38.7	38.7–43.0	43.0–57.3			
	PLI (£)	152	145	164	190	194	232	15.9	<0.001	0.196
	Milk yield (kg)	31	42	103	132	195	311	23.7	<0.001	0.004
	Fat (%)	0.07	0.08	0.07	0.07	0.05	0.05	0.009	<0.001	0.003
	Protein (%)	0.04	0.04	0.04	0.04	0.03	0.02	0.005	<0.001	0.009
Lactation 3		16.7–32.0	32.0–35.3	35.3–38.5	38.5–41.7	41.7–46.0	46.0–63.7			
	PLI (£)	138	146	137	177	184	203	20.3	<0.001	0.532
	Milk yield (kg)	–20	45	54	105	163	261	27.4	<0.001	0.090
	Fat (%)	0.07	0.06	0.06	0.05	0.03	0.02	0.010	<0.001	0.353
	Protein (%)	0.04	0.04	0.04	0.03	0.03	0.02	0.005	0.001	0.277
Lactation 4+		16.5–32.1	32.1–36.1	36.1–39.3	39.3–43.0	43.0–47.0	47.0–70.6			
	PLI (£)	94	94	115	124	135	150	15.7	<0.001	0.730
	Milk yield (kg)	–96	–61	15	0	61	146	26.6	<0.001	0.214
	Fat (%)	0.09	0.07	0.07	0.06	0.05	0.01	0.010	<0.001	0.036
	Protein (%)	0.04	0.04	0.04	0.04	0.03	0.02	0.005	0.001	0.088

## 4. Discussion

The overall objective of this study was to understand further individual cow intake, milk composition and efficiency responses when offered concentrates on an FTY basis. The farms involved in this study had an average herd size of 190 cows and average annual milk sales of 8780 kg/cow, which was substantially greater than the average values for the NI dairy herd during the year the study was conducted, namely 100 cows and 7429 kg of milk/cow per annum (DAERA, 2020). This is unsurprising, as an FTY approach to concentrate feeding is more likely to be adopted within higher yielding herds as concentrate feed levels are higher. Mean milk fat and protein percentages of cows in the study (4.12% and 3.26%, respectively) were similar to the NI average (4.07% and 3.31%, respectively: DAERA, 2020).

### 4.1. Diets Offered and Feed Intake

Diets offered on the farms involved in this study were typical of those in NI, with grass silage the predominant forage in the diet over the winter months (40–46% of total DMI). Maize silage and cereal silage were also offered on 17 of the 26 farms, comprising approximately 20% of total forge DMI on those farms during the study period. Although the proportion of alternative forages in the diet decreased across the milk yield groups in each lactation, this reflects the increase in total DMI as actual intakes of alternative forages remained relatively constant. While the average composition of the forages offered generally indicated they had a good nutritive value, there was considerable variability across farms, as demonstrated by the data range presented.

As the study involved an on-farm approach, all intakes were predicted, with the exception of intakes of the concentrate component of the diet obtained directly from the feeding systems on some farms. As cows within this study were offered concentrates on an FTY basis, it followed that concentrate DMI increased with increasing milk yield group. In contrast, while forage DMI decreased in lactation 4+ across milk yield groups (0.6 kg/day between the highest and lowest milk yield groups), forage DMI was unaffected by the milk yield group in lactations 2 and 3 while actually increasing in lactation 1. These results demonstrate that substitution of forage by concentrates, in general, was minimal to non-existent, which contrasts with the findings of a meta-analysis by Huhtanen et al. [6], which indicated a 0.47 kg reduction in forage DMI per kg extra concentrate consumed. However, Purcell et al. [5] also observed low substitution rates in a study examining FTY systems. The near absence of forage substitution observed lends support to the assumption commonly used when rationing cows on an FTY basis, namely that the basal diet is likely to sustain a relatively constant level of performance (i.e., a constant M+) for cows across a range of milk yield potentials.

Although total DMI was predicted within this study (mean for all lactations, 22.0 kg DM/cow day), comparable intakes in early lactation cows offered similar grass silage-based diets were recorded by Little et al. [4] and Purcell et al. [5] (22.3 and 22.4 kg per cow/day) which provide confidence in the current results. Furthermore, the 17%, 24% and 32% increase in total DMI between lactation 1 and each of lactations 2, 3 and 4+ was similar to the increases observed between lactations in a meta-analysis of 27 feeding studies involving grass silage-based diets (22%, 31% and 34%, respectively) [14]. Total DMI showed a linear increase with increasing milk yield group (and, as such, concentrate level) within each lactation, which is in contrast to most traditional studies examining the relationship between milk yield and concentrate intake, where total DMI normally follows a curvilinear response [15,16,17]. Thus, the current study highlights a key difference between traditional studies examining performance responses to concentrate feeding and studies examining an FTY approach. In the former, ‘balanced’ groups of cows of mixed yield potential are allocated to a number of different concentrate ‘level’ treatments. The curvilinear response at higher concentrate levels is a consequence of the inability of cows with a lower yield potential within these groups to respond to higher concentrate feed levels fully. In contrast, within an FTY approach, higher levels of concentrates are offered only to higher-yielding cows, and these have a greater intake potential, resulting in the observed linear response in total DMI. Therefore, the results from the current study support the findings of both Purcell et al. [5] and Little et al. [4], who noted that the increase in DMI with increasing milk yield was greater for cows offered concentrates on an FTY basis compared to a ‘flat rate’ basis.

In contrast to TMR feeding, in which the forage: concentrate ratio remains constant with each group of cows, the forage: concentrate ratio of individual cows offered concentrates on an FTY basis varies according to milk yield. For example, the mean concentrate proportion in the diet increased as the milk yield group increased, from between 40.3 to 42.7% in the lowest milk yield groups to between 52.6 to 56.6% in the highest milk yield groups. However, individual cows within the highest and lowest milk yield groups will have been offered diets with more extreme forage: concentrate ratios than the group average. Increasing concentrate proportions led to an increase in total diet starch content (from 12.6 to 15.0% DM) and total diet CP content (from 16.0 to 16.9% DM: mean within lowest and highest milk yield bands). Given that the quantity of alternative forage (maize silage and whole crop silage) in the diets remained relatively constant across bands, the increasing starch content of the diet was largely driven by increasing concentrate proportion in the diet. However, this effect was mitigated to some extent by the concentrate component of the diet offered on an FTY basis having a lower mean starch and CP content than the concentrate included within the basal rations.

### 4.2. Milk Production and Composition

On the majority of farms, the concentrate component of the diet offered on an FTY basis was offered at a feed rate of 0.45 kg concentrate (fresh) per kg milk. This commonly adopted feed rate assumes that 1.0 kg of concentrate contains approximately 11.5 MJ of ME (fresh) and that the production of 1.0 kg of milk requires approximately 5.2 MJ of ME. The higher feed rate adopted on two farms within this study is likely to reflect a desire to increase milk production further, while the lower feed rates adopted on five farms within the study is likely to reflect a desire to produce more milk from forage.

The increase in milk yield between the lowest and highest milk yield groups is partly due to the increase in PTA for milk (kg) between these extreme groups, namely 166, 280, 281 and 242 kg (lactations 1–4+, respectively). These genetic differences could account for an additional 332, 560, 562 and 484 kg milk/lactation between the extreme milk yield groups, or 1.1, 1.8, 1.8 or 1.6 kg additional milk/day during a 305-day lactation. Nevertheless, actual differences in milk yield between the extreme milk yield groups were 17.0, 21.6, 23.1 and 23.1 kg/day for lactations 1–4+, respectively, considerably greater than the values expected based on differences in PTA. These differences can be largely explained by the increase in concentrate proportion in the diet, total DMI, and diet starch and CP content across the milk yield group. However, given that concentrates were offered on an FTY basis, these factors alone do not explain how such differences arose initially. The most likely explanation is the effect of differing management systems in early lactation prior to the start of FTY. During the ‘concentrate build-up period’, concentrate inputs likely reflect the overall management system operating on each individual farm (e.g., ‘low’, ‘moderate’ or ‘high’ concentrate input), with that system likely determined to some extent by herd genetic potential. For example, when examining the concentrate DMI during the first month of lactation, farms offering higher levels of concentrate during month 1 post-calving also offered higher levels of concentrate during month 2–5 post-calving. Furthermore, a greater proportion of cows from higher-yielding herds were within higher groups, and vice versa for cows from lower-yielding herds. Differences in forage quality, concentrate type, and overall management will also have impacted performance at this time. Thus, differences in performance which arose in early lactation as a result of farm-specific management decisions will have placed cows on these farms on a specific milk yield ‘trajectory’, and with the introduction of FTY, higher-yielding cows will have moved to higher concentrate levels, which will drive higher yields and concentrate intakes within a repeated feedback loop.

In a previous AFBI study, milk fat concentration declined from 40.9 to 38.1 g/kg as the feed rate increased from 0.45 to 0.55 kg concentrate/kg milk [18], while an analysis of individual cow data within that study indicated that both milk fat and milk protein concentration decreased substantially with increasing concentrate level within the two feed rates (C. Ferris, unpublished data). Therefore, a key objective of the current study was to examine the impact of FTY concentrate feeding approaches on milk composition on commercial farms. This is especially important for farmers supplying milk for processing, where milk pricing mechanisms normally include either bonuses or deductions around a ‘base price’ according to milk composition.

In agreement with the findings of Purcell et al. [5], the milk fat in the current study showed a significant (*p* < 0.01) decrease between the lowest and highest milk yield groups (by 0.49, 0.67, 0.52 and 0.77 a percentage unit in lactations 1, 2, 3 and 4+, respectively). Part of this reduction, especially in multiparous cows, is likely to reflect the decreasing PTA for milk fat% with an increasing milk yield group. For example, the decrease in PTA for milk fat% observed between the lowest and highest concentrate levels could account for a reduction in milk fat of 0.04, 0.04, 0.10, and 0.16 of a percentage unit in lactations 1, 2, 3 and 4+, respectively. Thus, differences in cow genotype between the extreme concentrate treatments would account for up to 8, 6, 19 and 21 percent of the reduction in milk fat% in each of lactations 1, 2, 3 and 4+, respectively. The remaining ‘non-genetic’ component of the decrease in milk fat% is likely due to the ‘dilution effect’ of increased milk yields [19,20] and the effect of diet, although as the DMI prediction equation included fat:protein ratio, it is possible that this may have introduced an element of bias. Increasing concentrate levels have been associated with a reduction in milk fat% in many studies [7,16,17,21], with this primarily driven by the increasing starch content of the diet [22,23]. The increasing starch content was likely accompanied by a decrease in fibre content (although fibre content was not measured), particularly within the concentrate faction of the diet, which would have further exacerbated milk fat depression. Rapid rumen fermentation of concentrate starch can result in a fall in rumen pH [24], reduced microbial biohydrogenation of dietary unsaturated fatty acids, and greater ruminal production of trans-fatty acids (for example, trans-10, cis-12 CLA) [25,26]. These trans-fatty acids can inhibit milk fat synthesis in the mammary gland. While significant reductions in milk fat% were not observed in some studies until the concentrate proportion of the diet reached 0.56 [5] or even 0.70 [17], reduced milk fat% in the current study was observed at a concentrate proportion in the diet of less than 0.50.

Milk protein% also decreased across milk yield groups in each lactation 1, 2, 3 and 4+ (by 0.22, 0.36, 0.36 and 0.30 of a percentage unit, respectively). Part of this reduction (between 0.02 and 0.04 of a percentage unit) can likely be attributed to the decrease in PTA for milk protein% between the lower and higher milk yield groups. Thus, differences in cow genotype between the extreme concentrate treatments would account for up to 9, 11, 11 and 13 percent of the reduction in milk protein% in each of lactations 1, 2, 3 and 4+, respectively. Milk protein% is generally influenced by energy supply, particularly through the breakdown of starch to glucose and the associated increase in microbial protein synthesis [27]. Therefore, the increase in diet starch intake with increasing milk yield group would be expected to increase milk protein% [28], as has been observed in many studies [22,29,30]. The ‘non-genetic’ component of the reduction in milk protein% in the current study is therefore difficult to explain and may reflect a dilution effect associated with increased milk yield [19] and higher-yielding cows having a poorer energy balance [4]). It was not possible to measure cow BW or body condition score within this study, so the impact of the milk yield group on body tissue reserves was not examined. While an FTY approach is designed to supply concentrates to meet the theoretical energy requirements of each individual cow, in reality, the many assumptions that are involved in FTY calculations mean that this is unlikely to be the case. Nevertheless, previous studies observed that the range in energy balance experienced by cows offered concentrates on an FTY basis, particularly for higher-yielding cows, was smaller than for cows offered concentrates on a ‘group’ basis [4,5].

The reduction in PTA for both milk protein% and milk fat% with increasing milk yield group is likely to reflect sires used in higher-yielding herds having been selected with an increased focus on milk volume and with less attention paid to milk constituents. In scenarios where farms receive either a bonus or deduction on milk price, according to milk fat and protein percentages, a reduction in milk composition will reduce the value of each litre of milk produced. It is possible that more precision could be introduced into FTY systems by taking into account the milk composition of individual cows when allocating concentrates. For example, across all lactations, the mean GE content of milk within the lowest and highest milk yield groups was 3.3 and 3.0 MJ/kg, respectively (based on the equation published by AFRC, 1990 as described previously). Assuming an efficiency of lactation (kl) of 0.64 [31], the ME required to produce each kg of milk is calculated as 5.2 and 4.7 MJ for the lowest and highest milk yield groups, respectively. Assuming a concentrate ME content of 11.5 MJ/kg fresh (13.0 MJ/kg DM, as adopted in the current study), then appropriate feed rates would be 0.45 and 0.40 kg concentrate (fresh)/kg milk for the lowest and highest milk yield groups, respectively). This suggests that different feed rates may be justified depending on the composition of milk produced. Nevertheless, in a recent study, no overall improvement in production efficiency was observed when such an approach was adopted [32].

### 4.3. Feed Use Efficiency

Feed use efficiency, normally defined as the ratio of ‘output to input’, is of increasing importance due to environmental pressures, the need to improve resource use efficiency, and the potential to improve farm profitability [33], provided performance, health, fertility and longevity is not compromised. Improving NUE can reduce N excretion from dairy systems and contribute to reduced N losses to the atmosphere as ammonia [34] and nitrous oxide [35] and waterways [36]. While it is recognised that all efficiency measures presented use estimated DMI as the denominator and, as such, are dependent on the accuracy of the estimate of DMI, it is still worthwhile examining these while bearing in mind the limitations. Across all lactations, NUE increased with increasing milk yield group, despite an increase in total diet CP levels and a reduction in milk protein. While NUE normally improves with decreasing diet protein levels [37,38,39], the opposite effect in the current study can be attributed to the dilution of protein requirements for maintenance with increasing milk N output in milk. Mean NUE in this study (0.30, across all lactations) was similar to that of Huhtanen et al., namely 0.28 [39].

As with NUE, both ECM/DMI and milk energy/ME intake increased with increasing milk yield group across all lactations. While increasing genetic merit for milk yield is normally associated with improvements in gross feed use efficiency [40], there is little evidence that cows with greater milk yield have better metabolic efficiency for milk production (kl) than lower-yielding cows [41]. Instead, the increases in efficiency with increasing concentrate levels in the current study can be largely attributed to ‘dilution of maintenance energy requirements’ with increasing milk yields and perhaps increased mobilisation of body tissue reserves for milk production, as discussed previously.

While concentrate DMI/milk yield represents a ‘crude’ efficiency factor, it is a useful on-farm metric which can be determined using readily available farm data. In general, at any given milk yield, a higher value suggests a lower efficiency of concentrate use and a reduced reliance on forage in the diet. Within the current study (across all lactations), the mean concentrate use efficiency was 0.30, similar to the value of 0.31 determined by Wilkinson (2011) for the UK dairy sector [42]. When expressed per kg milk produced, this value did not differ across milk yield groups in multiparous cows, decreasing from 0.31 to 0.30 in primiparous cows. In contrast, when expressed on an ECM yield basis, there was a small reduction in efficiency (increasing values) with an increase in the milk yield group in all lactations, reflecting the decreasing fat and protein concentration of milk (and associated lower ECM yield, compared to milk yield) at higher concentrate levels. Given the large range in concentrate levels between milk yield groups, a greater increase in concentrate use efficiency values might have been expected. That this was not observed likely reflects the fact that concentrates were offered on an FTY basis.

## 5. Conclusions

Responses, when concentrates are offered on an FTY basis, are very different from those observed within traditional concentrate response studies. Total DMI continued to increase with increasing milk yields, with little evidence of forage substitution by concentrates. The lack of forage substitution observed lends support to the assumption that a basal diet will sustain a common milk yield across a range of performance levels. Milk fat and protein concentration decreased with increasing milk yield, which is partly attributed to cow genetics, diet, and dilution effects. Differences in cow genotype between the two extreme milk yield treatment groups were calculated to be responsible for up to 21% of the variation in milk fat concentration and up to 13% of the variation in milk protein concentration observed between these treatments. Cows with greater milk yields had improved N and energy use efficiency due to the dilution of maintenance as milk yields increased. However, concentrates offered per kg of ECM increased with increasing milk yield. While higher levels of concentrate feeding sustain higher milk yields, the reduction in milk composition, and the subsequent impact on the value of each kg of milk value produced, may impact the economics of offering concentrates according to an FTY approach.

## Figures and Tables

**Table 1:** Chemical composition of conserved forages and concentrates offered (mean, minimum and maximum) to cows throughout the study period (based on samples collected at each farm visit).

			Mean ± SD	Range	
				Min	Max
Conserved forages					
	Grass silage				
		Oven dry matter (g/kg)	318 ± 39	211	393
		Crude protein (g/kg DM)	139 ± 9	122	155
		Neutral detergent fibre (g/kg DM)	425 ± 25	395	480
		Metabolisable energy (MJ/kg DM)	11.2 ± 0.5	10.0	12.1
		pH	3.96 ± 0.21	3.70	4.66
		Ammonia nitrogen (g/kg total N)	81 ± 24	60	190
		Predicted acid load (meq/kg DM)	718 ± 30	700	834
	Maize silage				
		Oven dry matter (g/kg)	334 ± 65	267	439
		Crude protein (g/kg DM)	86 ± 7	81	97
		Metabolisable energy (MJ/kg DM)	11.4 ± 0.5	10.5	11.9
		Starch (g/kg DM)	293 ± 69	211	387
		Neutral detergent fibre (g/kg DM)	413 ± 40	341	568
	Whole crop cereal silage				
		Oven dry matter (g/kg)	399 ± 95	252	584
		Crude protein (g/kg DM)	84 ± 12	64	107
		Metabolisable energy (MJ/kg DM)	10.0 ± 0.7	9.2	11.1
		Starch (g/kg DM)	244 ± 84	46	344
Concentrates					
	Offered on a feed-to-yield basis				
		Crude protein (g/kg DM)	193 ± 10	155	250
		Starch (g/kg DM)	229 ± 27	135	313
	Offered within the basal ration				
		Crude protein (g/kg DM)	219 ± 27	100	345
		Starch (g/kg DM)	247 ± 45	129	418

## Data Availability

None of the data were deposited in an official repository.
